# Neuromolecular basis of faded perception associated with unreality experience

**DOI:** 10.1038/s41598-018-26382-9

**Published:** 2018-05-23

**Authors:** Keita Yokokawa, Takehito Ito, Keisuke Takahata, Harumasa Takano, Yasuyuki Kimura, Masanori Ichise, Yoko Ikoma, Ayako Isato, Ming-Rong Zhang, Kazunori Kawamura, Hiroshi Ito, Hidehiko Takahashi, Tetsuya Suhara, Makiko Yamada

**Affiliations:** 1 Department of Functional Brain Imaging, National Institute of Radiological Sciences, National Institutes for Quantum and Radiological Science and Technology, 4-9-1 Anagawa, Inage-ku, Chiba Chiba, 263-8555 Japan; 2 Tohoku University Graduate School of Medicine, 2-1 Seiryo-machi, Aoba-ku, Sendai, Miyagi 980-8575 Japan; 3 Integrative Brain Imaging Center, National Center of Neurology and Psychiatry, 4-1-1 Ogawa-Higashi, Kodaira, Tokyo 187-8551 Japan; 4 Department of Molecular Imaging and Theranostics, National Institute of Radiological Sciences, National Institutes for Quantum and Radiological Science and Technology, 4-9-1 Anagawa, Inage-ku, Chiba, Chiba 263-8555 Japan; 5 Department of Radiopharmaceuticals Development, National Institute of Radiological Sciences, National Institutes for Quantum and Radiological Science and Technology, 4-9-1 Anagawa, Inage-ku, Chiba, Chiba 263-8555 Japan; 6 Department of Radiology and Nuclear Medicine, Fukushima Medical University, 1 Hikariga-oka, Fukushima, Fukushima 960-1295 Japan; 7 Department of Neuropsychiatry, Kyoto University School of Medicine, 54 Shogoin Kwaramachi, Sakyo-ku, Kyoto, Kyoto 606-8507 Japan; 8 Group of Quantum and Cellular Systems Biology, QST Advanced Study Laboratory, National Institutes for Quantum and Radiological Science and Technology, 4-9-1 Anagawa, Inage-ku, Chiba, Chiba, 263-8555 Japan

## Abstract

Perceptual changes in shape, size, or color are observed in patients with derealization symptoms; however, the underlying neural and molecular mechanisms are not well understood. The current study explored the relationship between neural activity associated with altered colorfulness perception assessed by fMRI and striatal dopamine D_2_ receptor availability measured by [^11^C]raclopride PET in healthy participants. Inside an fMRI scanner, participants performed the saturation adaptation task, where they rated how much vivid/faded visual objects looked like real/unreal ones using a visual analog scale. We found that participants experienced greater unreality when they perceived fadedness than vividness despite physically identical saturation. The combined fMRI and PET analyses revealed that the faded perception-related activities of the dorsolateral prefrontal and parietal cortex were positively correlated with striatal D_2_ receptor availability. This finding may help to understand the neuromolecular mechanisms of faded perception associated with feeling unreal in derealization symptoms.

## Introduction

Derealization is an abnormal subjective experience in which the external world appears strange or unreal. DSM-V defines the perceptual alteration in derealization as “experiences of unreality or detachment with respect to surroundings (e.g., individuals or objects are experienced as unreal, dreamlike, foggy, lifeless, or visually distorted)^1^”. Derealization is observed not only in a wide range of psychiatric disorders, such as depersonalization disorder, schizophrenia, depression and anxiety disorder^2–4^, but also in healthy people. Population surveys revealed that derealization as well as depersonalization that is the experience of unreality in sense of the self^5^ were experienced in healthy subjects with the prevalence of about half in a sample population of college students^6,7^.

Perceptual alterations of shape, size, or color in derealization have been less studied, and thus little is known about the neural mechanisms of altered visual perception associated with feeling unreal. In the field of vision studies, however, there is ample experimental evidence of perceptual changes of visual objects induced by adaptation techniques in healthy individuals^8^. In particular, the perceptual alteration of colorfulness is produced by saturation adaptation, in which, after being exposed with highly saturated stimuli, the following object looks faded, while the same object looks vivid after perceiving low saturation stimuli^9^. Thus, application of saturation adaptation in fMRI may reveal the neural activities associated with subjective faded/vivid perception toward physically identical saturation objects.

Dopamine is a key modulator for learning and cognitive control, but also for visual experience. It has been shown that dopaminergic activation facilitates visual perceptual performance^10–13^, improves the cortical neuronal signal-to-noise ratio (SNR)^14–16^, and enhances the aftereffects of visual adaptation^17^. A recent study using positron emission tomography (PET) further revealed that the dopamine D_2_ receptor availability in striatum was positively correlated with subjective visual experience^18^. Thus, it is speculated that striatal dopaminergic transmission may affect how visual information, including the aftereffects of saturation adaptation, is processed in cerebral cortex.

Here, we set out to assess the subjective colorfulness and unreality experience of visual objects, and to examine the relationships between the neural representation of subjective colorfulness and unreality, measured by fMRI, and striatal D_2_ receptor availability, measured by [^11^C]raclopride PET, in healthy subjects.

## Results

### Behavioral results

**Figure 1:**
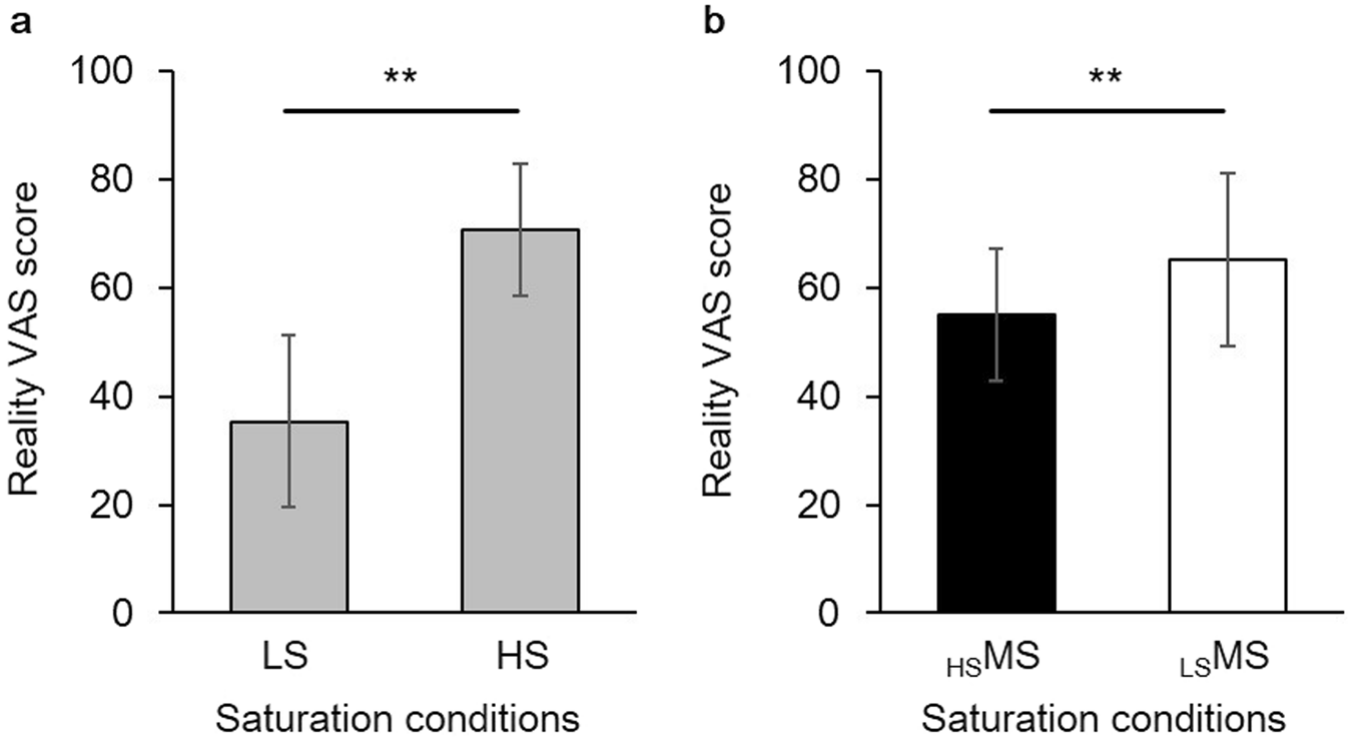
Behavioral data. (**a**) There were statistical differences in reality ratings between objective faded condition (LS) and objective vivid condition (HS), and (**b**) between subjective faded condition (_HS_MS) and subjective vivid condition (_LS_MS). ^**^p < 0.001. Error bars indicate s.d.

The reality ratings of low saturation stimuli (LS, objective faded condition) (35.4 ± 15.9) were significantly lower than those of high saturation stimuli (HS, objective vivid condition) (70.6 ± 15.9) (*n* = 14, paired *t*-test, *t*_(13)_ = −9.1, *p* < 0.001, Fig. 1a), showing that participants perceived stronger unreality for the physically low saturation stimuli. In case of physically identical middle saturation stimuli (MS), the reality ratings of middle saturation stimuli preceded by high saturation stimuli (_HS_MS, subjective faded condition) and middle saturation stimuli preceded by low saturation stimuli (_LS_MS, subjective vivid condition) were 55.0 ± 11.0 and 65.2 ± 8.7, respectively. The ratings of the subjective faded condition (_HS_MS) were significantly lower than those of the subjective vivid condition (_LS_MS) (*n* = 14, paired *t*-test, *t*_(13)_ = −4.8, *p* < 0.001, Fig. 1b), indicating that participants perceived stronger unreality for the subjective faded condition (_HS_MS) than the subjective vivid condition (_LS_MS) despite their saturation levels being physically identical.

### fMRI results

**Figure 2:**
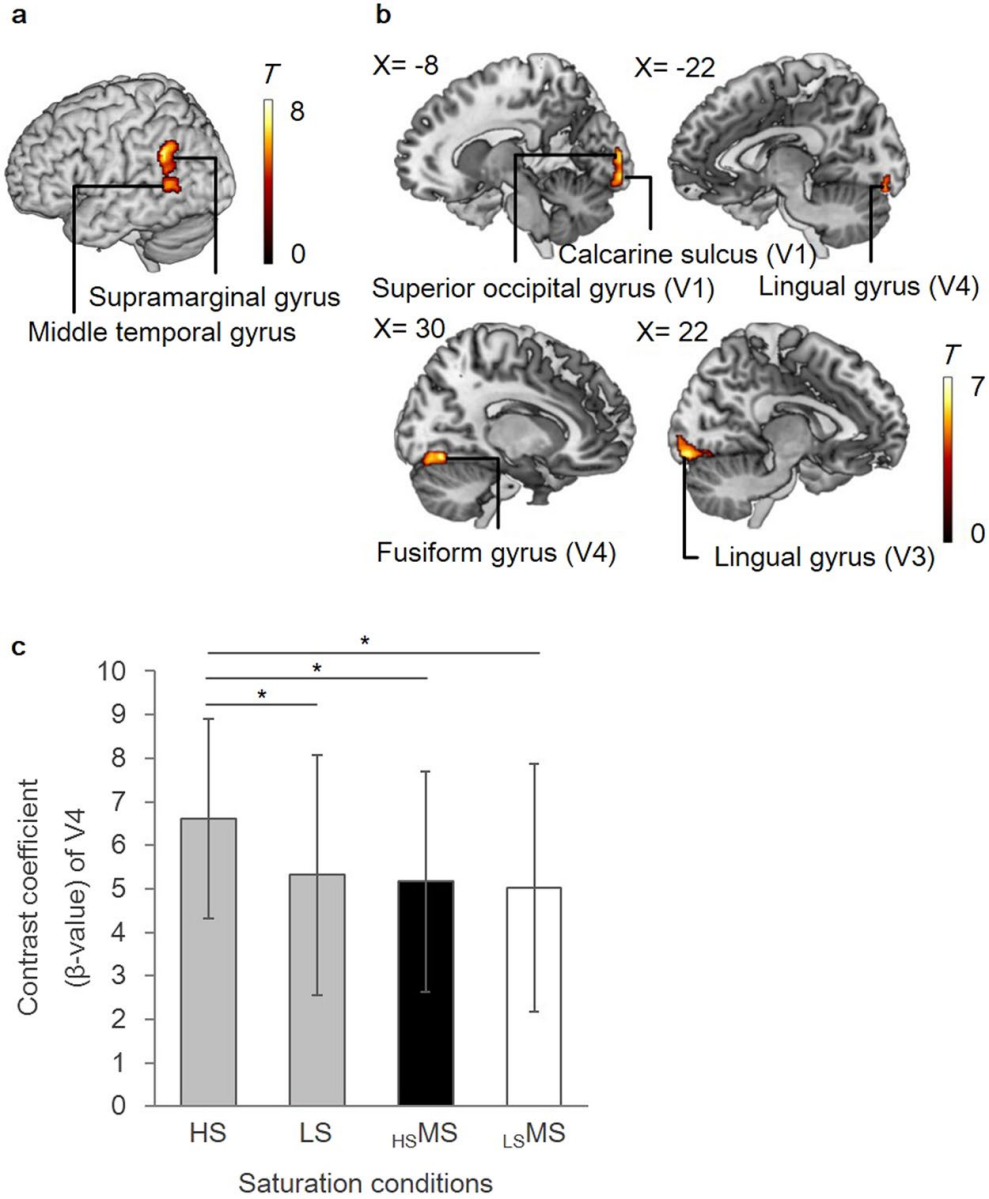
Regions associated with objective faded and vivid conditions. (**a**) The contrast of objective faded condition (LS) versus objective vivid condition (HS) revealed increased activities in the left parietal cortex (voxel level of *p* < 0.001; cluster level of *p* < 0.05, FDR corrected). (**b**) The contrast of objective vivid condition (HS) versus objective faded condition (LS) yielded activities in the bilateral occipital cortex (voxel level of *p* < 0.001; cluster level of *p* < 0.05, FDR corrected). (**c**) The bilateral V4 activity of objective vivid condition (HS) was higher than any other conditions. ^*^p < 0.05, Bonferroni corrected. Error bars indicate s.d.

The contrast of the objective faded condition (LS) versus the objective vivid condition (HS) recruited the activity of parietal cortex (voxel level of *p* < 0.001; cluster level of *p* < 0.05, FDR corrected, Fig. 2a, Table 1), while the contrast of the objective vivid condition (HS) versus the objective faded condition (LS) revealed significant activations in visual areas including bilateral V4, known as the color processing regions (voxel level of *p* < 0.001; cluster level of *p* < 0.05, FDR corrected, Fig. 2b, Table 1). We further compared the ß-values extracted from V4 (MNI = −30/−84/−8 and 34/−80/−8 at 4-mm sphere, based on Rottschy *et al*.^19^) in the contrast of color pictures (HS, LS, _HS_MS, _LS_MS) versus scramble images. This revealed that the average activity of right and left V4 was significantly higher for the objective vivid condition (HS) than any other conditions (HS versus LS: *p* = 0.018; HS versus _HS_MS: *p* = 0.028; HS versus _LS_MS: *p* = 0.048, all *p*-values after Bonferroni corrected for multiple comparison, Fig. 2c). Thus, the effect of physically different saturation was observed in parietal cortex for the objective faded condition, whereas the objective vivid condition recruited more activation in visual processing areas. The comparison of physically identical MS did not yield any significant activations, but only at the liberal threshold (voxel level of *p* < 0.001; *k* > 20)^20^, the contrast of the subjective faded condition (_HS_MS) versus the subjective vivid condition (_LS_MS) showed increased activations in the right thalamus, left amygdala and left inferior frontal cortex (Supplementary Fig. S1, Supplementary Table S1). The contrast of the subjective vivid condition (_LS_MS) versus the subjective faded condition (_HS_MS) did not show any activated regions at this liberal threshold.

**Table 1:** Brain regions associated with the contrast of objective conditions.

Brain region	L/R	BA	MNI coordinate			T values (peak level)	Cluster size	p-values (voxel level)	p-values (cluster level)
			**x**	**y**	**z**				
**LS versus HS**									
Supramarginal gyrus	L	40	−50	−48	28	7.83	620	<0.001	<0.001
Middle temporal gyrus	L	39	−52	−56	8	5.79	—	<0.001	—
**HS versus LS**									
Fusiform gyrus	R	—	28	−66	−14	6.28	341	<0.001	=0.001
Lingual gyrus (V3)	R	18	22	−88	−12	6.12	—	<0.001	—
Fusiform gyrus (V4)	R	18	30	−74	−14	5.51	—	<0.001	—
Calcarine gyrus (V1)	R	17	20	−96	−2	5.06	—	<0.001	—
Calcarine gyrus (V2)	L	17	−8	−96	−8	6.83	214	<0.001	=0.004
Superior occipital gyrus (V1)	L	17	−12	−96	4	6.08	—	<0.001	—
Calcarine gyrus (V1)	L	18	−6	−96	4	5.98	—	<0.001	—
Superior occipital gyrus	L	18	−10	−96	10	5.81	—	<0.001	—
Calcarine gyrus (V1)	L	17	−14	−96	−4	5.78	—	<0.001	—
Lingual gyrus (V4)	L	18	−22	−86	−14	5.06	—	<0.001	—
Lingual gyrus (V3)	L	18	−16	−88	−12	4.69	—	<0.001	—

R: right, L: left, BA: Brodmann’s area. Significant clusters obtained from the contrast of “LS vs. HS” and “HS vs. LS” (voxel level of *p* < 0.001; cluster level of *p* < 0.05, FDR corrected).

### PET and fMRI results

**Figure 3:**
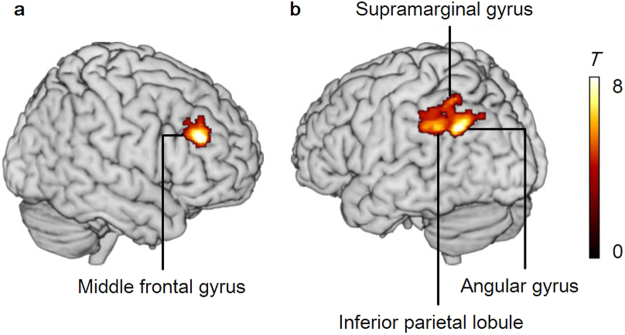
Subjective fadedness regions associated with dopamine D_2_ receptor availability. The D_2_ *BP*_ND_ values of executive striatum were positively correlated with (**a**) activity of middle frontal gyrus (voxel level of *p* < 0.05, FWE corrected; cluster level of *p* < 0.05, FDR corrected), and (**b**) activity of inferior parietal lobule (voxel level of *p* < 0.001; cluster level of *p* < 0.05, FDR corrected) for the contrast of subjective faded condition (_HS_MS) versus subjective vivid condition (_LS_MS).

The mean D_2_ *BP*_ND_ values of three subdivisions of left and right striatum are shown in Supplementary Table S2. Multiple regression analyses were conducted to examine the association between the neural activities of the subjective faded condition in the contrast of _HS_MS versus _LS_MS and D_2_ *BP*_ND_ in three sub-regions of left and right striatum. The D_2_ *BP*_ND_ value of left executive striatum was positively correlated with the activity of the right dorsolateral prefrontal cortex (middle frontal gyrus, BA 46, voxel level of *p* < 0.001; cluster level of *p* < 0.05 (FDR corrected)) and the left parietal cortex (including left angular gyrus (BA 39), left inferior parietal lobe (BA 40), and left superior parietal lobule (BA 7); voxel level of *p* < 0.001; cluster level of *p* < 0.05 (FDR corrected)) (Fig. 3, Table 2), but there were no regions associated with D_2_ *BP*_ND_ values of other subdivisions of striatum or in the contrast of the subjective vivid condition (_LS_MS) versus the subjective faded condition (_HS_MS). Analyses of the contrasts of physically different saturation levels (LS versus HS) did not yield any significant regions associated with D_2_ *BP*_ND_ values of any subdivisions of striatum, but the D_2_ *BP*_ND_ value of left executive striatum in the objective faded condition (LS) versus the objective vivid condition (HS) contrast correlated with the left parietal cortex (left inferior parietal lobule (BA 40)) at a marginally significant level (cluster level of *p* = 0.08, FDR corrected), which overlapped with the left parietal cortex found in subjective faded condition (overlapped region was presented in Supplementary Fig. S2).

**Table 2:** Brain regions of unreal perception correlated with striatal D_2_ *BP*_ND_ of left executive striatum.

Brain region	L/R	BA	MNI coordinate			*T* values (peak level)	Cluster size	*p*-values (voxel level)	*p*-values (cluster level)
			x	y	z				
Frontal lobe									
Middle frontal gyrus	R	46	46	40	28	10.44	265	<0.001	=0.033
Middle frontal gyrus	R	9	32	36	38	4.49	—	<0.001	
Middle frontal gyrus	R	9	44	30	38	4.44	—	<0.001	
Parietal lobe									
Angular gyrus	L	32	−30	−56	32	9.05	689	<0.001	<0.001
Angular gyrus	L	39	−34	−62	36	7.40	—	<0.001	—
Inferior parietal lobule	L	40	−42	−44	36	5.78	—	<0.001	—
Supramarginal gyrus	L	40	−50	−40	34	5.78	—	<0.001	
Inferior parietal lobule	L	40	−42	−58	54	5.76	—	<0.001	—
Inferior parietal lobule	L	40	−50	−54	50	5.14	—	<0.001	
Angular gyrus	L	40	−42	−56	44	4.57	—	<0.001	
Inferior parietal lobule	L	40	−56	−46	44	4.55	—	<0.001	
Inferior parietal lobule	L	40	−54	−48	48	4.54	—	<0.001	

R: right, L: left, BA: Brodmann’s area. Significant clusters obtained from the contrast of _HS_MS vs. _LS_MS with left striatal D_2_*BP*_ND_ as a covariate (voxel level of *p* < 0.001; cluster level of *p* < 0.05, FDR corrected).

## Discussion

The current study examined the dopaminergic neural mechanisms of perceptual changes in colorfulness associated with the unreality experience in healthy subjects. We revealed that subjective fadedness was accompanied with unreality experience, and that the individual differences of dopamine D_2_ receptor availability in executive striatum, as measured by PET, was positively associated with dorsolateral prefrontal and parietal activities related to subjective faded perception, as measured by fMRI.

Previous vision study observed that the subjective experience of colorfulness changed following the adaptation to the different levels of saturation^9^. By applying this adaptation methodology in the current study, we further revealed that the unreality experience was increased toward the visual object adapted to a high level of saturation (subjective faded condition), in contrast to the identical object adapted to a low level of saturation (subjective vivid condition). This behavioral result suggests that subjective fadedness accompanies the subjective experience of unreality.

We discovered that the individual differences of dopamine D_2_ receptor availability in executive striatum were positively associated with activities of the right middle frontal gyrus (BA46), a part of the dorsolateral prefrontal cortex (DLPFC), and the left parietal cortex for the subjective faded condition. Dopamine has been proposed as playing a key role in prediction and the specification of own expectation^21,22^, which helps to build adaptive internal models of the body and the world. This scheme is proposed particularly in motor control^23^ but may also be employed for the formation of visual world, based on the current findings as well as several previous studies that demonstrated the influences of dopamine on the enhancement of visual perceptual performance and acuity^11,13^. In addition, impaired color perception has frequently been reported in patients with Parkinson’s disease that is the case of dopaminergic dysfunction^24^, and their abnormal color vision can be reversed by treatment with levodopa that is an amino acid precursor of dopamine^25^. Our previous study using [^11^C]raclopride and _L_-[β-^11^C]DOPA revealed that postsynaptic D_2_ receptor binding potentials were inversely related with presynaptic dopamine synthesis^26^. Therefore, the current finding indicates that individuals with lower endogeneous dopamine synthesis show higher activity in the frontoparietal network for subjective faded perception, and the lower dopamine may invade to build internal models of the predicted world, observed as the unreality feeling in the current study. Enhancement of dopamine is supposed to treat depersonalization theoretically^27^, and the current study may provide the possible role of dopamine on unreality experience associated with faded perception.

Frontal and parietal cortex, where the former is anatomically connected with executive striatum^28^ and both are functionally connected with striatum^29^, compose the top-down attentional control system^30,31^. Previous neuroimaging studies of depersonalization disorder patients observed increased activities in the frontal and parietal cortex^32–35^, and proposed that the emotional numbing or detachment in these patients was associated with the hyperactive top-down control system that inhibits emotional responses. DLPFC is also associated with the experience of presence in a virtual reality environment^36^, where the higher activity in DLPFC was associated with the lower experience of presence and reality^37^. Moreover, derealazation is observed as ictal phenomena in patients with temporal and parietal lobe epilepsy^38^. Taken together with our findings, these results indicate that the increased neural activity in frontal and parietal cortex plays a role for unreality feeling associated with subjective fadedness.

Furthermore, over the last two decades, a strong association of frontal and parietal activity and visual awareness has been established^39^. A number of studies have shown that frontoparietal activity is associated with changes in the contents of visual consciousness^40^. Taking together both the observations of the striatal dopaminergic transmission and the proposed frontoparietal system, it could be speculated that the unreality experience based on subjective faded perception is influenced by the interaction between striatal dopaminergic transmission that plays a role in broadcasting the expectation-based sensory information to the cortex and the frontoparietal network, which in turn are necessary for conscious perception of fadedness and for the top-down allocations based on the subjective faded perception induced by the experimental settings of the current study.

Lastly, it should also be noted that while the objective vivid condition recruited more activation in visual areas including the color processing region, these regions were not modified by subjective faded and vivid conditions or by individual differences of dopamine D_2_ receptor availability in striatum.

One of the major limitations of the current study is that the findings of aftereffects may be explained not at neural levels but rather at retinal levels. Although studies indicate that saturation adaptation is a product of the brain^41^, we still cannot deny the possibility of retinal adaptation. In particular, it is known that dopamine receptors in retina have impacts on visual adaptation^42^ and that Parkinson’s disease has also shown some evidence of retinal dysfunction^43^. Thus, future study will hopefully clarify the association and dissociation between retinal and neural visual information processing, which are relevant to the emergence of unreality experience, together with the role of dopaminergic transmission. Second, the current data strongly suggest the involvement of striatal dopamine transmission in the neural activity of subjective faded and unreality experience, but do not determine the precise functional role of dopamine nor clarify the nature of the underlying neural processes. One line of future experimentation will be to apply the pharmacological approaches that have been used to study the impact of dopamine transmission on visual perceptual performance. At last, [^11^C]raclopride has equal affinity for D_2_ and D_3_ receptors. However, the previous study showed putamen and caudate are almost filled with all D_2_ receptors^44^. Executive striatum is composed of mostly putamen and caudate; thus, the current finding is regarded to reflect D_2_ receptor availability rather than D_3_ receptors.

In conclusion, the current findings highlight the neural underpinnings of the unreality experience induced by subjective faded perception, and the individual variabilities of striatal dopamine D_2_ receptor availability were associated with the function of the frontoparietal network in a healthy population. Although these results are only correlational and a causal relation will still need to be established, we demonstrated that striatal D_2_ receptor availability is positively related to the neural networks of faded perception and unreality experience. The findings may suggest the underlying mechanisms of the relatively neglected phenomena of derealization, and help in the consideration of possible treatment targets for this population.

## Methods

### Participants

Seventeen right-handed healthy male subjects (mean age = 23.9 ± 5.4 (mean and standard deviation) years) participated in the study. All participants had no history of neurologic or psychiatric disorder, and they were not taking any medications that could interfere with task performance or PET/fMRI data. Three participants were excluded from the analysis owing to insufficient quality of data recording: two were excluded due to excessive head movements in fMRI scan (more than 1 voxel), and one was excluded due to inadequate understanding of the task procedure. Therefore, the final sample comprised fourteen participants (mean age = 23.4 ± 4.2). Each subject underwent fMRI and PET scans. All participants provided written informed consent before participating in the study, which was approved by the Ethics and Radiation Safety Committee of the National Institute of Radiological Sciences in accordance with the ethical standards laid down in the 1964 Declaration of Helsinki and its later amendments.

### PET Procedures

#### Data acquisition

After intravenous injection of [^11^C]raclopride (228.4 ± 8.1 MBq with specific activity of 158.4 ± 53.5 GBq/µmol), three-dimensional dynamic images were acquired on a PET camera (Eminence SET-3000GCT/X, Shimadzu, Kyoto, Japan) for 60 min. All PET images were reconstructed by filtered back-projection method (Gaussian filter, kernel 5 mm; the reconstructed in-plane resolution was 7.5 mm in full-width at half-maximum; voxel size: 2 × 2 × 2.6 mm) corrected for attenuation, randoms, scatter and head motion.

#### Data analysis

The PET image analysis was performed using PMOD software 3.5 (PMOD Technologies Ltd. Zurich, Switzerland). Quantitative analysis was performed using the multilinear reference tissue model (MRTM2)^45^, which provides parametric images of the binding potentials (*BP*_ND_). All parametric images were spatially normalized to the MNI152 standard space based on the transformation parameters from the MR images estimated by DARTEL toolkit of SPM8. A connectivity-based probabilistic atlas of the striatum^28^ was applied to the spatially normalized parametric images to extract weighted-means of *BP*_ND_ values among corresponding voxels for the three sub-regions of left and right striatum (limbic, executive, and sensorimotor). Contribution of the bilateral sub-regions to the total striatal volume was 20, 49, and 24%, respectively^28^.

### fMRI Procedures

#### Stimuli

We used 20 pictures of flowers from the image library (Datacraft Inc. Hokkaido, Japan). The level of saturation was adjusted to yield high saturation, middle saturation and low saturation levels, using Adobe Photoshop software (Adobe Systems Inc. California, USA). The level of saturation for high saturation stimuli (HS) was increased by 79.0% (±7.7%) from middle saturation stimuli (MS), and that for low saturation stimuli (LS) was decreased by 73.3% (±11.5%) from MS based on our pilot experiment.

#### Task

The target MS images were presented after both HS and LS to induce different saturation perception and a sense of reality for the targets. “MS presented after HS” (_HS_MS, subjective faded condition) was supposed to induce low saturation perception, and “MS presented after LS” (_LS_MS, subjective vivid condition) was supposed to induce high saturation perception. Our pilot examination showed that saturation perception was significantly lower for the subjective faded condition (_HS_MS) than the subjective vivid condition (_LS_MS) (*n* = 8, paired *t*-test, *t*_(7)_ = −3.40, *p* = 0.012, Supplementary Fig. S3a), and saturation perception was positively correlated with reality perception (all *p*-values ≤ 0.001, Supplementary Fig. S3b). Thus, perception of higher (or lower) saturation in the subjective faded condition (_HS_MS) was associated with higher (or lower) reality in the subjective vivid condition (_LS_MS) for the current fMRI experiment.

The task instruction was given as follows: “The purpose of the present study is to evaluate the quality of 4 cameras. A series of flowers taken by 4 different cameras will be presented on the screen, and your task is to evaluate how real each picture looks.” In each trial, after a fixation (mean = 3.5 seconds, jittered between 2 and 6 seconds), a picture was presented for 8 seconds, and participants rated how much they felt reality using a visual analog scale (VAS), ranging from 0 (feeling no reality) to 100 (feeling strong reality).

**Figure 4:**
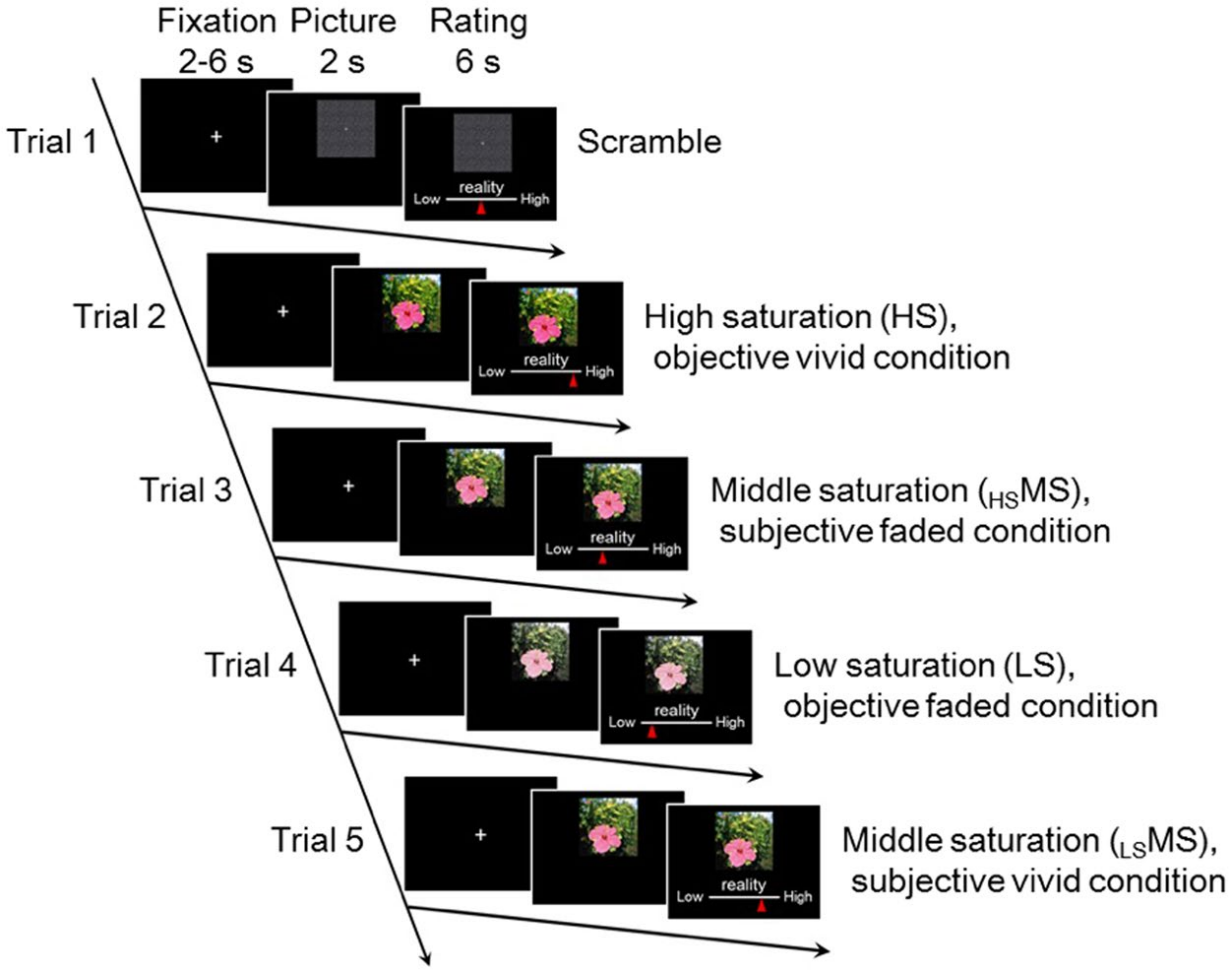
Task design. Each block contains five trials. At the beginning of a block, the scramble image was presented, and then a series of flower pictures was presented in the following order: high saturation (HS), middle saturation (_HS_MS), low saturation (LS) and middle saturation (_LS_MS). MS trials were always preceded by HS and LS trials. The orders of HS and LS were counterbalanced. These images were created only for the purpose of presentation.

The order of the trials was as follows: objective vivid condition (HS), subjective faded condition (_HS_MS), objective faded condition (LS), and subjective vivid condition (_LS_MS) (Fig. 4). The objective vivid condition (HS) and the objective faded condition (LS) were counterbalanced across trials. A trial with the gray-scaled scrambled image was always presented before the 4 saturation trials, and the participants were asked to move a curser to any place using VAS. Thus, five trials (one scrambled and four pictures) were grouped as a set, and one run consisted of 5 sets (25 trials). Participants performed 4 runs (100 trials) in total.

#### Behavioral data analysis

We examined the differences of reality ratings between the objective vivid condition (HS) and the objective faded condition (LS) and between the subjective faded condition (_HS_MS) and the subjective vivid condition (_LS_MS) to test the effects of physically different saturation and of saturation adaptation on reality experience, respectively. Two-tailed paired *t*-tests were performed using SPSS (IBM Corp. Released 2011. IBM SPSS Statistics, Version 20.0. Armonk, NY: IBM Corp.). *p* < 0.05 was considered statically significant.

#### fMRI data acquisition

Functional imaging was performed with a GE 3.0-T Excite system to acquire gradient echo T2*-weighted echo planar images with blood oxygenation level-dependent contrast. Each volume comprised 35 transaxial contiguous slices with a slice thickness of 3.8 mm to cover almost the whole brain (flip angle, 90°; echo time, 25 ms; repetition time, 2,000 ms; matrix, 64 × 64; interleaved acquisition). A high-resolution T1-weighted magnetization-prepared gradient echo sequence (124 contiguous axial slices; 3D spoiled-GRASS sequence; slice thickness, 1.5 mm; flip angle, 30°; echo time, 9 ms; repetition time, 22 ms; matrix, 256 × 192) was also collected for spatial normalization and localization.

#### fMRI preprocessing

Functional image analysis was performed using Statistical Parametric Mapping (SPM8; www.fil.ion.ucl.ac.uk/spm). EPI images were corrected for slice timing and rigid head motion. Head motion parameters were examined, and we confirmed that all trials had less than one voxel of translation and 2.0° of rotation in each participant. T1 images were segmented and spatially normalized to the same stereotaxic space by using the diffeomorphic anatomical registration through exponentiated Lie algebra (DARTEL) algorithm^46^. The voxel values of segmented and normalized gray matter images were modulated by the Jacobian determinants obtained from nonlinear normalization steps. After normalization, images were spatially smoothed using a Gaussian kernel with a full-width at half-maximum of 6 mm. High-pass temporal filtering (filter width of 128 seconds) was also applied to the data.

#### fMRI data analysis

The first-level analysis contained five regressors of conditions (HS, LS, _HS_MS, _LS_MS, and a scrambled image) using a general linear model (GLM). For each condition, the whole period from the onset of the stimuli (8 seconds) was modeled and was convolved with SPM8’s standard canonical hemodynamic response function. Six realignment parameters and two derivatives were used as covariates. All artifacts in fMRI time-series data were detected and corrected with Robust WLS Toolbox in SPM8^47^.

For the group analyses, the individual contrast images of LS versus HS and of _HS_MS versus _LS_MS were analyzed in a random-effects model. Each model included the mean-centered D_2_ *BP*_ND_ values of each striatal sub-region as a covariate of interest to examine how the individual differences of D_2_ *BP*_ND_ were associated with the neural responses of unreality experience. Resulting p values were corrected for multiple comparisons. We report here a voxel level of *p* < 0.001 (uncorrected), a cluster level of *p* < 0.05 (corrected by false discovery rate (FDR)), as significant results^48^. Brain figures were created using MRIcron (http://people.cas.sc.edu/rorden/mricron/index.html)^49^.

#### ROI analysis

To compare the activity of bilateral V4, we constructed the bilateral V4 mask image (MNI = −30/−84/−8 and 34/−80/−8 at 4-mm sphere, based on Rottschy *et al*.^19^, and extracted the contrast coefficient (ß-value) of each condition. These regions corresponded to V4 areas defined by the SPM Anatomy Toolbox (v2.2). One-way analysis of variance (ANOVA) with Bonferroni’s multiple comparison post-test was used to determine statistical significance (*p* < 0.05) using SPSS.

## Electronic supplementary material

Supplementary Information
