# Supplementary material for: Neuromolecular basis of faded perception associated with unreality experience
Source: Sci Rep. 2018 May 23;8:8062. doi: 10.1038/s41598-018-26382-9 (PMC5966381; doi:10.1038/s41598-018-26382-9)
Supplement: Supplementary file 1 — Supplementary Information [file 41598_2018_26382_MOESM1_ESM.docx]

Supplementary Information for

Neuromolecular basis of faded perception associated with unreality experience

Keita Yokokawa,^1,2^ Takehito Ito,^1^ Keisuke Takahata,^1^ Harumasa Takano,^1,3^ Yasuyuki Kimura,^1^ Masanori Ichise,^1^ Yoko Ikoma,^4^ Ayako Isato,^1^ Ming-Rong Zhang,^5^ Kazunori Kawamura,^5^ Hiroshi Ito,^6^ Hidehiko Takahashi,^7^ Tetsuya Suhara,^1^ and Makiko Yamada^1,8*^

^1^ Department of Functional Brain Imaging, National Institute of Radiological Sciences, National Institutes for Quantum and Radiological Science and Technology, Chiba, Chiba, Japan

^2^ Tohoku University Graduate School of Medicine, Sendai, Miyagi, Japan

^3^ National Center of Neurology and Psychiatry, Kodaira, Tokyo, Japan

^4^ Department of Molecular Imaging and Theranostics, National Institute of Radiological Sciences, National Institutes for Quantum and Radiological Science and Technology, Chiba, Chiba, Japan

^5^ Department of Radiopharmaceuticals Development, National Institute of Radiological Sciences, National Institutes for Quantum and Radiological Science and Technology, Chiba, Chiba, Japan

^6^ Department of Radiology and Nuclear Medicine, Fukushima Medical University, Fukushima, Fukushima, Japan

^7^ Department of Neuropsychiatry, Kyoto University School of Medicine, Kyoto, Kyoto, Japan

^8^ Group of Quantum and Cellular Systems Biology, QST Advanced Study Laboratory, National Institutes for Quantum and Radiological Science and Technology, Chiba, Chiba, Japan

* Corresponding Author: yamada.makiko@qst.go.jp

**Supplementary Figures**


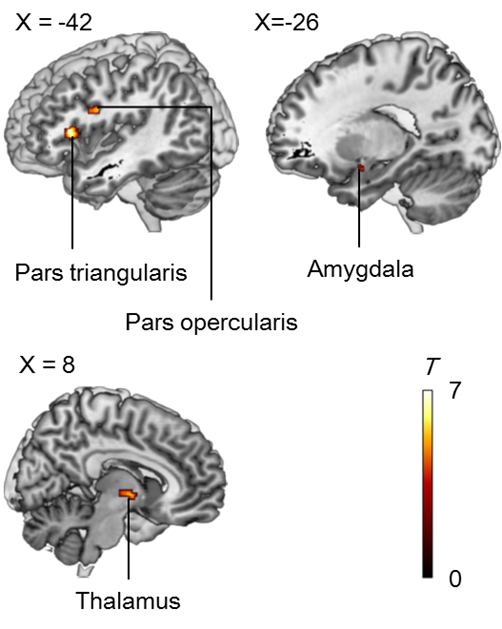


**Supplementary Figure S1**. These regions were more activated when perceiving _HS_MS than _LS_MS conditions (voxel level of *p* < 0.001, *k* > 20), indicating that they are associated with subjective faded perception.


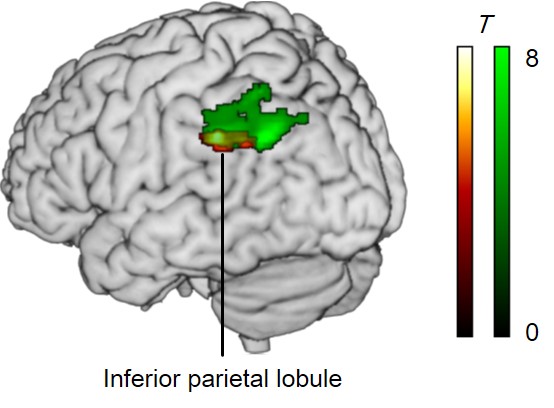


**Supplementary Figure S2.** Regions associated with dopamine D_2_ receptor availability for the contrast of LS versus HS (red, voxel level of *p* < 0.001; cluster level of *p* = 0.08, FDR corrected) and the contrast of _HS_MS versus _LS_MS (green, voxel level of *p* < 0.001; cluster level of *p* < 0.05, FDR corrected). Areas of common activation include the left inferior parietal lobule.


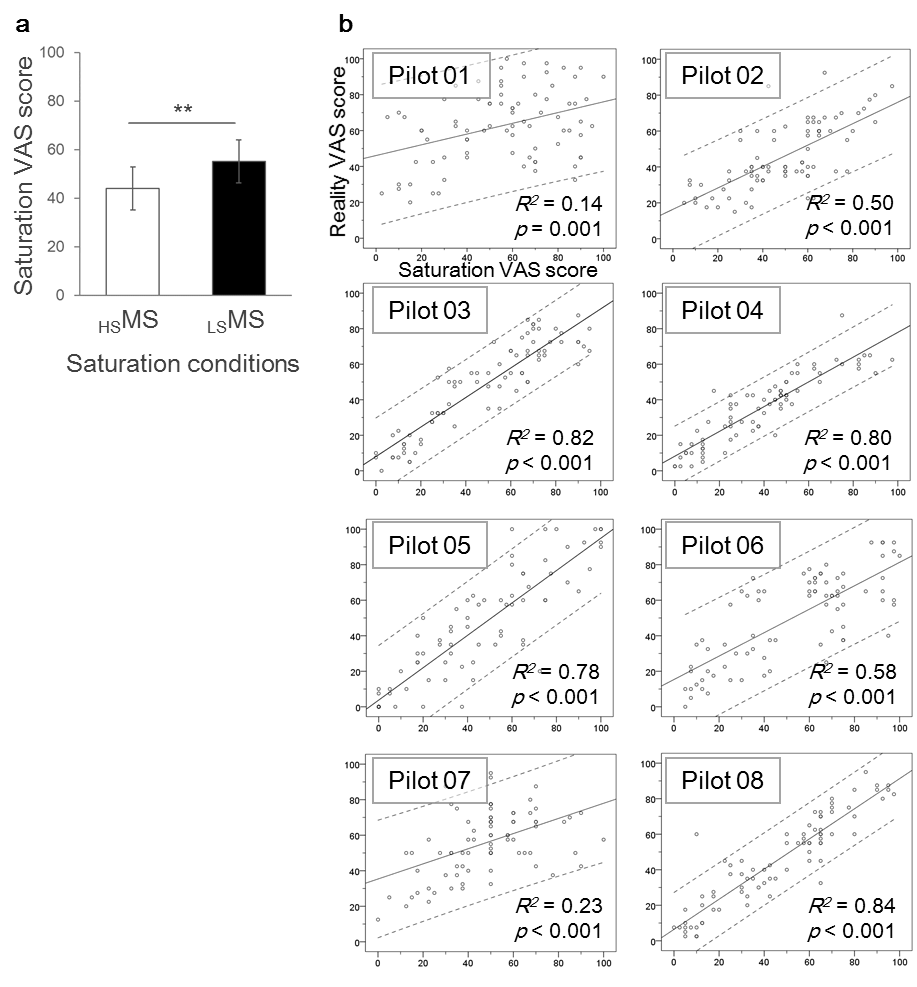


**Supplementary Figure S3.** (**a**) Mean saturation ratings and standard deviations of MS conditions in the pilot study are plotted. It was also confirmed that the perceived saturation of the MS image was influenced by the levels of saturation (HS or LS) immediately before presenting (*n* = 8, paired t-test, *t*_(7)_ = -3.40, *p* = 0.01). Thus, MS following HS induced lower saturation perception compared to MS following LS. (**b**) Each perceived plot indicates mean VAS score of individual stimulus. Scatter plots depict the relationship between saturation VAS score and reality VAS score for individual stimulus images (80 images) in each pilot subject (*n* = 8). Positive correlation is observed in the reality evaluation for subjective visibility when the perceived saturation is higher (*n* = 8, simple regression analysis, *R^2^* range: 0.14-0.84, all *p*-values ≤ 0.001). Solid line denotes linear regression line and dashed line denotes 95% confidence interval.

**Supplementary Tables**

Supplementary Table S1. Brain regions associated with subjective faded condition

| **Brain region** | **L/R** | | **BA** | | **MNI coordinate** | | | | | ***T* values**  **(peak level)** | **Cluster size** | **p-values**  **(voxel level)** |
| --- | --- | --- | --- | --- | --- | --- | --- | --- | --- | --- | --- | --- |
|  |  |  |  |  | **x** | | **y** | | **z** |  |  |  |
| Frontal lobe | | | | | | | | | | | | |
| Pars triangularis | L | 45 | | -42 | | 24 | | 6 | | 7.6 | 54 | <0.001 |
| Pars opercularis | L | 9 | | -42 | | 2 | | 24 | | 5.55 | 31 | <0.001 |
| Thalamus | R | - | | 8 | | -6 | | -2 | | 5.63 | 35 | <0.001 |
| Amygdala | L | - | | -26 | | 0 | | -16 | | 4.23 | 26 | <0.001 |
| R: right, L: left, BA: Brodmann’s area.  Significant clusters obtained from the contrast of “_HS_MS vs. _LS_MS” (voxel level of *P* < 0.001, *k* > 20). | | | | | | | | | | | | |

Supplementary Table S2. Dopamine D_2_ receptor binding potential (*BP_N_*_D_) of [^11^C]raclopride

|  | *BP*_ND_ | CV (%) |
| --- | --- | --- |
| Limbic striatum |  |  |
| - Right Limbic striatum | 2.20 ± 0.30 | 14 |
| - Left Limbic striatum | 2.27 ± 0.29 | 13 |
| Executive striatum |  |  |
| - Right Executive striatum | 2.44 ± 0.20 | 8 |
| - Left Executive striatum | 2.37 ± 0.22 | 9 |
| Sensory-motor striatum |  |  |
| - Right Sensory-motor striatum | 2.58 ± 0.30 | 12 |
| - Left Sensory-motor striatum | 2.53 ± 0.29 | 12 |
| *BP*_ND_: mean and standard deviation, CV: coefficient of variation. | | |
